# Actions and Roles of FSH in Germinative Cells

**DOI:** 10.3390/ijms221810110

**Published:** 2021-09-18

**Authors:** Kaiana Recchia, Amanda Soares Jorge, Laís Vicari de Figueiredo Pessôa, Ramon Cesar Botigelli, Vanessa Cristiane Zugaib, Aline Fernanda de Souza, Daniele dos Santos Martins, Carlos Eduardo Ambrósio, Fabiana Fernandes Bressan, Naira Caroline Godoy Pieri

**Affiliations:** 1 Department of Surgery, Faculty of Veterinary Medicine and Animal Sciences, University of São Paulo, São Paulo 01001-010, Brazil; kaiana.recchia@usp.br (K.R.); fabianabressan@usp.br (F.F.B.); 2 Department of Veterinary Medicine, School of Animal Sciences and Food Engineering, University of Sao Paulo, Pirassununga 13635-900, Brazil; amanda_soares_jorge@usp.br (A.S.J.); laisvpessoa@usp.br (L.V.d.F.P.); ramonbotigelli@alumni.usp.br (R.C.B.); vanessa.zugaib@usp.br (V.C.Z.); daniele@usp.br (D.d.S.M.); ceambrosio@usp.br (C.E.A.); 3 Department of Pharmacology, Institute of Biosciences, São Paulo State University (UNESP), Botucatu 18618-970, Brazil; 4 Department Biomedical Science, Ontary Veterinary College, University of Guelph, Guelph, ON N1G 2W1, Canada; adesou08@uoguelph.ca

**Keywords:** gonadotrophin, germ cell line, reproduction

## Abstract

Follicle stimulating hormone (FSH) is produced by the pituitary gland in a coordinated hypothalamic–pituitary–gonadal (HPG) axis event, plays important roles in reproduction and germ cell development during different phases of reproductive development (fetal, neonatal, puberty, and adult life), and is consequently essential for fertility. FSH is a heterodimeric glycoprotein hormone of two dissociable subunits, α and β. The FSH β-subunit (FSHβ) function starts upon coupling to its specific receptor: follicle-stimulating hormone receptor (FSHR). FSHRs are localized mainly on the surface of target cells on the testis and ovary (granulosa and Sertoli cells) and have recently been found in testicular stem cells and extra-gonadal tissue. Several reproduction disorders are associated with absent or low FSH secretion, with mutation of the FSH β-subunit or the FSH receptor, and/or its signaling pathways. However, the influence of FSH on germ cells is still poorly understood; some studies have suggested that this hormone also plays a determinant role in the self-renewal of germinative cells and acts to increase undifferentiated spermatogonia proliferation. In addition, in vitro, together with other factors, it assists the process of differentiation of primordial germ cells (PGCLCs) into gametes (oocyte-like and SSCLCs). In this review, we describe relevant research on the influence of FSH on spermatogenesis and folliculogenesis, mainly in the germ cell of humans and other species. The possible roles of FSH in germ cell generation in vitro are also presented.

## 1. Introduction

Follicle stimulating hormone (FSH) and luteinizing hormone (LH) are gonadotropins essential for proper reproduction and fertility in both females and males; specifically, they are key players in germ cell formation [1,2]. In females, several essential oocyte formation processes are triggered by gonadotropins FSH and LH, mainly through the stimulation of granulosa cell proliferation, antrum formation in secondary ovarian follicles, growth and maturation of antral follicles, and estradiol production, resulting, together, in folliculogenesis, oogenesis, oocyte meiotic maturation, and oocyte competence [3,4]. In males, these hormones initiate their function at puberty, when FSH acts in the induction of spermatogenesis and LH induces androgen production by Leydig cells [1]. FSH activates Sertoli cell proliferation, first during fetal development, and continuing at puberty. In adult life, FSH acts through its receptors on the Sertoli cells in spermatogenesis, germ cell survival, and male fertility [5,6]. Furthermore, through these cells, this hormone indirectly regulates spermatogonial stem cells (SSCs) and creates the adequate environment for germinative cells proliferation and differentiation [7]. Additionally, they regulate other physiological activities that have biological effects on the hypothalamus, pituitary, and reproductive organs [2,8,9,10].

FSH and LH are secreted as part of the hypothalamic–pituitary–gonadal (HPG) axis. Gonadotrophin-releasing hormone (GnRH) is produced by the hypothalamus, reaches the anterior pituitary through local circulation, stimulates gonadotrophic cells to produce and release the gonadotrophins into the circulation of the organism, and stimulates the anterior pituitary [1]. FSH is a heterodimeric glycoprotein composed of two non-covalently bound α and β subunits [11]. The α-subunit is encoded by a single gene that is common to FSH, LH, human chorionic gonadotropin (hCG), and thyroid-stimulating hormone (TSH), although the β-subunit is FSH-specific [12]. The FSH β-subunit (FSHβ) acts in its target cells (granulosa and Sertoli cells) when connected to its follicle-stimulating hormone receptor (FSHR), localized in the cellular membrane [13,14]. FSHR belongs to the family of G protein-coupled receptors (GPCRs), which act by the activation of various signaling pathways (cAMP/PKA, PKC/MAPK, and Ca^+^/CaMKII) [14,15,16,17,18,19]. However, FSHR can also be found in extra-gonadal organs and tumorous tissues, including different types of cancer, tumor vessels, endothelial cells, osteoclasts, human umbilical vein endothelial cells, monocytes, the liver, and in a population of stem cells called very small embryonic-like stem cells (VSELs) [5,20,21,22,23,24,25,26,27,28,29,30,31,32,33].

Several reproduction disorders are associated with absent or low FSH secretion, which may result from mutations of the FSHβ or in the FSHR, and from disorders unrelated to mutations, but related to the FSH signaling pathways [34,35]. In this regard, the condition called hypogonadism if often classified into three categories: primary hypogonadism (hypergonadotropic, resulting from a primary testicular disorder); secondary hypogonadism, which is congenital or acquired (hypogonadotropic, presenting deficiencies in the hypothalamus and/or pituitary); mixed hypogonadism (affecting both testes and the hypothalamus/pituitary). These latter two are determined by alterations in the HPG system, resulting in impaired testicular function and leading to the condition called hypogonadotropic hypogonadism (HH) [36,37].

In men, primary hypogonadism is associated with low testosterone production and normal or high levels of the FSH and LH hormones. The possible causes include testicular injury, congenital anorchidism, cryptorchidism, mumps orchitis, tumor, testicular trauma, genetic defects (Klinefelter syndrome and gonadal dysgenesis), Sertoli-cell-only syndrome, chemotherapy, radiation treatment, alcohol abuse, and autoimmune syndromes. In Klinefelter syndrome, the pituitary-gonadal function may be normal during childhood and even during early puberty; however, in adult life, the FSH and LH levels increase and testosterone decreases [38,39].

Secondary and mixed HH present low or inappropriate LH and FSH levels and sex steroids, which are associated with lower sperm quantity. HH can result from genetic disorders (e.g., lack of GnRH production in Kallmann syndrome) or may have acquired causes such as drugs, infiltrative or infectious pituitary lesions, hyperprolactinemia, encephalic trauma, pituitary/brain radiation, exhausting exercise, and abusive alcohol or illicit drug intake [40,41]. Patients with HH caused by hypothalamic disorders have been treated with exogenous pulsatile GnRH therapy, associated or not with gonadotropin treatment, aiming at spermatogenesis stimulation and production of competent spermatozoa, whereas FSH treatment is still in the experimental phase in patients presenting idiopathic normogonadotropic infertility and oligozoospermia [1,40,42]. 

In women, the increase in FSH level is a usual indication of premature ovarian failure, leading to menopause occurrence before the age of 40 years, possibly resulting from genetic causes; however, most cases are idiopathic. Turner syndrome is a genetic disease caused by the loss of an X chromosome (XO karyotype) or chimeric monosomic (X0/XX) karyotype, also possibly leading to premature ovarian failure or even primary amenorrhea, underdeveloped ovaries (streak ovaries), and high FSH levels [43,44].

Conversely, in women presenting ovulatory dysfunction, FSH treatment has been recommended for decades; for example, using human chorionic gonadotropin (hCG) and human menopausal gonadotropin (hMG) to stimulate the growth and ovulation of the dominant follicle in patients presenting anovulatory infertility. Novel therapies using biotechnology have been introduced; for example, recombinant gonadotrophin, which is purer than urinary-derived gonadotropins and shows better clinical efficiency [35,45,46]. Recombinant human FSH (rhFSH) has been shown to be efficient in patients with polycystic ovary syndrome (PCOS), which is anovulatory, in stimulating follicular growth [47,48,49]. Different strategies using FSH supplementation, alone or combined with a variety of pharmaceuticals, have been developed to be used for ovulation induction and assisted reproductive therapy, including the stimulation of multifollicle ovulation for embryo transfer [50]. In cattle, the stimulation of superovulation is routinely used to obtain the maximum number of viable embryos; usually, this is caused by FSH treatment based on recombinant hormone [51,52,53,54,55] or, historically, pituitary extracts [55].

In domestic animals, alterations in the FSH level are poorly described. A recent study in dogs showed that HH might occur in dogs (both sexes) with primary adrenocortical insufficiency (PAI), and HH is suspected to occur in dogs with concurrent polyendocrine diseases [56]. Other studies were conducted with genetically modified animals such as mice and pigs, aiming to identify specific genes to study the HH phenotype. In mice and pigs, knockout of the kisspeptin gene or its receptor resulted in the HH phenotype, and animals failed to initiate puberty. Male mice models presented small testes and failed sperm production, reduced production of steroids, and the absence of the development of secondary sexual characteristics. These females models of HH have failed to have normal ovarian follicular maturation and pregnancy [57,58,59].

In the past decade, FSH’s actions in reproductive physiological or pathological conditions have been more precisely studied, in both males and females, using genetic analysis approaches. Interestingly, mutations in the FSHβ sequence have already been reported as a cause for infertility in males, whereas FSHβ polymorphisms in females seem to affect the basic level of FSH in different ways, affecting the ovarian activity, either due to a positive response to exogenous gonadotropin administration or to alterations of the phenotype in several disfunctions such as PCOS and premature ovarian failure, for example [2,4,43,44,60,61,62,63,64,65,66]. 

Mammalian infertility may be resultant from different physiopathology-related disorders, as discussed before, and, interestingly, these have been associated with immunological alterations and even autoimmune failure. In females, the immune system may be activated against ovarian antigens, in general directed against FSH (anti-FSH), more specifically, against the β-chain form. The anti-FSH populations modulate the recognition and binding of FSH to FSHR, and it may have a pathological influence on ovarian function; thus, immunosuppressive treatment would be an option for patients with high concentrations of anti-FSH [67]. Anti-FSH has been predominantly present in patients with endometriosis and PCOS; however, it remains unknown if anti-FSH is the cause of infertility [67,68,69].

It has been reported that, during fertile life, women are more likely to present autoimmune diseases than men, and such observation appears after menopause, premature ovarian failure (POF), and other ovarian failure disorders, probably due to high FSH levels leading to a decrease in B lymphocytes and CD4, and other immune related dysregulation [70,71]. Indeed, the impacts of gonadotrophins in the immune system have been mainly reported in women; however, the precise relation of hormonal level and immunological function is still scantily described.

Apart from these important recent advances in the understanding of FSH’s roles and actions in the reproductive pathophysiology, few studies have explored the role of FSH in reproductive development and the influence of FSH on germinative cells in vivo and in vitro. Such an understanding is vital to obtaining new insights into the roles of FSH in special, new assisted biotechnologies. This review discusses the most relevant research on mechanisms through which FSH is involved in germ cells during all reproductive life. In addition, we present the use of FSH in the induction of germ cells and germ-cell-like cells in vitro.

## 2. The Role of FSH in Reproductive Development: Fetal and Neonatal Periods

**Figure 1:**
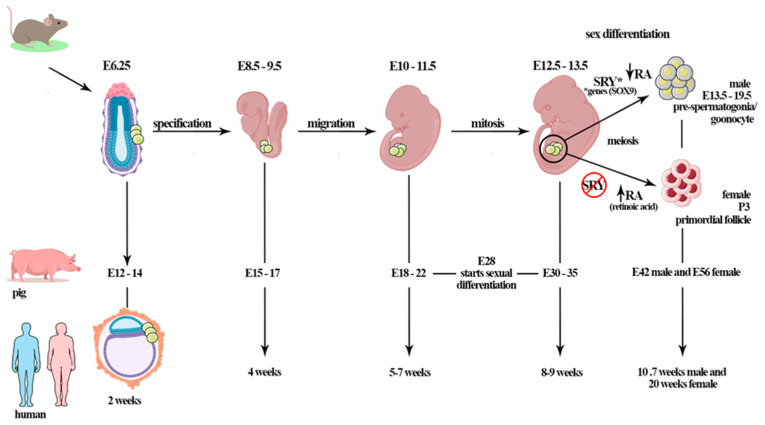
The mammalian gametogenesis process starts after the primordial germ cells (PGCs) complete the migration process to the gonadal ridge. The PGCs migration window is specific for each mammal species (e.g., ~E8–9.5 days for mice, ~E15–17 days for pigs, ~E25–30 days for cows, and ~4 weeks for humans). During PGCs colonization, the gonads are undifferentiated and, morphologically, are apparently identical (bipotential period). In porcine, gonadal differentiation starts at 28 days of gestation; however, complete differentiation is observed at E30–35 days of gestation. By E42, males have gonocytes in differentiated gonads; in females, the first primordial follicle is observed at E56 d.p.c in the fetal ovary. In mice, the germ cells of embryos at day E12.5 are morphologically undifferentiated; however, initiation of sex differentiation occurs at E13.5 days; the gonocytes are found from E13.5 until birth. In humans, PGCs colonize the genital ridge at 5–7 weeks (~E37), oogonia proliferation occurs until 10 weeks when the cells enter meiotic prophase, and primordial follicles are observed at 20 weeks of gestation. Sex determination is orchestrated by the SRY gene on the Y chromosome. *The expression of the SRY gene promotes testis formation through the activation of other genes (testicular genes, e.g., other factors and genes such as SOX9) during sex differentiation. The retinoic acid (RA) plays important role when PGCs enter meiosis. The female germ cells start meiosis after RA action, differently from males, in which meiosis pathway is inhibited by the RA-degrading enzyme CYP26B1.This period is gonadotropin-independent, although Sertoli cells expresses FSHR, that starts to act after birth.

In mammals, gametogenesis starts after the primordial germ cells (PGCs) complete the migration process to the gonadal ridge. The PGCs migration window is specific for each mammal species (e.g., ~E8–9.5 days for mice, ~E17 days for rabbits, ~E15–17 days for pigs, ~E25–30 days for cows, and ~4 weeks for humans) [72,73,74,75]. During PGC colonization, the gonads are undifferentiated and morphologically similar. This period is therefore called the bipotential stage of the gonadal ridge, where it develops and differentiates in the testis or ovaries [76] (Figure 1).

Sex differentiation is centrally orchestrated by the existence or absence of central *SRY* gene expression, which is located on the Y chromosome. The *SRY* gene is expressed in somatic cells subpopulations of undifferentiated gonads (mouse E10.5–12 d.p.c), and stimulates these cells to develop into Sertoli cells [77,78,79,80]. The expression of the *SRY* gene promotes testis formation through the activation of testicular-related genes, repressing ovarian fate [81]. However, studies have reported that XX males of different species (human, dog, mouse, and other mammals) who lack SRY present the male phenotype with normal Sertoli cells, suggesting that activation of other factors and the expression of genes such as *SOX9* are important for sex differentiation [81,82,83,84]. The expression of *SOX9*, for example, induces the differentiation of cells from the bipotential gonadal ridge into Sertoli cells, consequently contributing to the testis [76]. The SOX9 protein directly activates the transcription of at least some Sertoli-cell-specific genes, including the gene encoding anti-Mullerian hormone (AMH). The *SOX9* gene is not on the Y chromosome, but is expressed in males in most vertebrates, unlike *SRY*, which is found only in mammals [81].

In domestic animals such as pigs, morphological gonadal differentiation starts at 28 days of gestation, and complete differentiation is observed at 30–35 days of gestation (unpublished data [85]), at the fifth to sixth week in humans, and between E10.5 and 12 d.p.c, in mice, where clustered PGCs are distributed across the ovary in which the cortex and medulla cannot be distinguished at this stage [85]. In dogs, Souza et al. [86] reported the initiation of sexual differentiation at 35 days of gestation (middle trimester).

In mice, the germ cells of embryos at day E12.5 are morphologically undifferentiated; however, it is possible to observe differences between the somatic cells of ovaries and testes at E13.5 days, at the initiation of sex differentiation [87,88,89]. Afterward, female germ cells enter meiotic prophase I and begin to differentiate in oocytes. In humans, the PGCs colonize the genital ridge during the sixth week (~E37), and oogonia proliferation occurs until the tenth week, when the cells enter the meiotic prophase [90]. In bovines, oogonia was observed in the fetal ovary at D50–80 days of gestation [91].

During differentiation of the ovary before birth, PGCs proliferate and differentiate into oogonia and oocytes; later, these oocytes are enclosed by one layer of somatic cells called pre-granulosa cells, developing the primordial follicles when starting meiosis. Importantly, endocrine and paracrine factors delivered from oocytes act on the follicular cells, promoting multiple cell signaling pathways and driving differentiation. During follicular formation, FSHRs are expressed in some species, however, little is known about the factors responsible for its expression at such an early period. In rodents, the expression of FSHR increases with development, and its expression induces primary follicle formation and follicular development through the preantral stage [92]. In female pigs, the FSH level increases between days 75 and 90–103 p.c., before the emergence of primary follicles in the fetal ovary (106 p.c.) [93,94]. Therefore, FSH seems to be linked to the follicular process and primordial-to-primary follicle transition. In addition, gonadotropin-independent mechanisms are active in this phase, revealing that factors produced within the ovary and/or arriving via extrinsic pathways, such as activin [95], estrogens, and nerve growth factor [96], increase FSHR. FSHR has been reported in the fetal ovaries of cows, hamsters, baboons, and pigs [94,97,98].

In rats, it has been reported that the neonate ovary is insensitive to gonadotropins because it lacks FSH and LH receptors. However, the presence of FSHR and the functionality of the receptor were shown on days 5–7 after birth. In addition, on day 7 of post-natal development in rats, FSH-stimulated estradiol production was observed [44,99,100,101].

In mammalian males, during fetal life, PGCs migrate to the fetal testis, where they differentiate in gonocytes [102,103] (Figure 1). Firstly, PGCs and mesenchymal stem cells (MSCs), which are the precursors of Sertoli cells, form the seminiferous cord. At this stage, PGCs generate the gonocytes that remain centrally placed, surrounded by immature MSCs-derived Sertoli cells. In mice, gonocyte development occurs before the functional maturity of the pituitary gland, and then is not regulated by the gonadotrophins [104,105,106]. In vitro, a study on fetal and neonatal testes of rats showed that gonocytes survival is not influenced by FSH treatment [107]. However, in vitro culture of testes cells from three-day-old rats showed that FSH, combined with other factors such as follistatin, may mediate the maturation of gonocytes into spermatogonia [108].

During fetal life, the cells most influenced by FSH are Sertoli cells through the FSHR localized on cellular membranes and associated with G protein [1]. These receptors were reported in humans for the first time in testicular tissues at 8–16 weeks, with action starting after birth [109,110]. In Rhesus monkeys, Sertoli cells express FSHR early in the development at 19–22 weeks of gestation [110]. In rats, FSHR was detected in fetal and neonatal testis at E17.5–19.5 days of gestation, but at low expression levels [111].

Before puberty, FSH and FSHR pathway signaling regulates the proliferation of Sertoli cells and the number of these cells present in adult life [112]. For example, in rodents, FSH stimulates the proliferation of Sertoli cells in fetal and neonatal life, and it defines the number of these cells at puberty [113], different from Rhesus monkeys, in which the proliferation of Sertoli cells occurs, especially in the peripubertal phase. During these periods, FSH controls the proliferation of Sertoli cells by activating cyclic adenosine monophosphate/protein kinase A (cAMP/PKA) mitogen-activated protein kinase (ERK1/2), and phosphatidylinositol 3-kinase (PI3K)/serine/threonine protein kinase B (PKB/AKT)/mechanistic target of rapamycin (mTORC1) pathways, and increasing the transcription of c-Myc, hypoxia-inducible factor 2 alpha (*HIF2*), and cyclin D1 (*CCND1*) [112,114,115].

## 3. FSH in the Adult Phase: Folliculogenesis and Spermatogenesis

### 3.1. Females: FSH Influences Folliculogenesis

**Figure 2:**
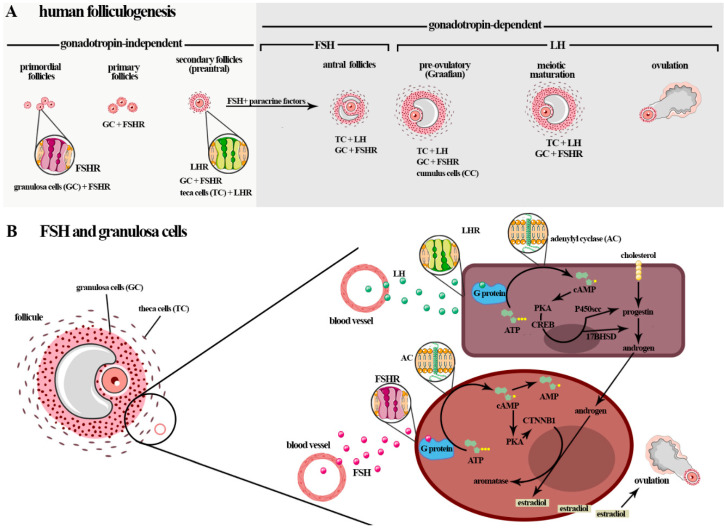
(**A**) Initially, the follicular growth that begins during gestation is gonadotropin-independent, although granulosa cells are influenced by FSH. The post-natal phases are gonadotrophin-dependent, and FSH influences the transition of the GCs of the larger antral follicles to the ovulatory follicles. After birth, folliculogenesis, a highly regulated process, can be classified into three phases: follicle growth, transition and maturation, and ovulation. In humans and mice, five stages of follicular development are described: primordial follicles, primary follicles, secondary follicles (preantral), antral follicles, and preovulatory follicles (Graafian). The phase of follicle transition from the preantral stage to the early antral stage and follicle growth and maturation is dependent on stimulus by FSH and LH (gonadotropin-dependent phase), which play an obligatory role in follicle differentiation, selection, and survival. (**B**) FSH actions in folliculogenesis, including in the induction of GCs proliferation and stimulation of GCs in the estradiol production by aromatase (CYP19A1) conversion of the androgens produced by theca cells (i.e., dehydroepiandrosterone (DHEA), androstenediol, androstenedione, and testosterone) from cholesterol in responding to LH (steroidogenesis). The interaction of the FSH-FSHR localized in the membrane surfaces of GCs activates adenylyl cyclase and stimulates the proliferation of CG cells by activating the cyclic adenosine monophosphate/protein kinase A (cAMP/PKA), mitogen-activated protein kinase/extracellular signal-regulated kinase (MAPK/ERK), and PI3K/Akt pathways. In particular, cAMP/PKA promotes the phosphorylation of cyclic AMP response element-binding (CREB) protein and other proteins that promote an increase in the expressions of genes that encode the growth factors and proteins involved in steroid hormone production and cellular growth. This gonadotropin can impede the apoptosis of GCs via the PI3K/Akt pathway.

In adult females of different mammalian species, ovarian function is regulated by gonadotropins and intra-ovarian factors (e.g., steroids, growth factors, and cytokines). They act in folliculogenesis, enabling follicle development and the differentiation of the granulosa cells (GCs) of the ovarian follicle [116]. Folliculogenesis, a highly regulated process, refers to ovarian follicle growth and differentiation, and can be classified into three main phases: initial follicle growth, transition from pre-antral to early antral phase, and growth and maturation until ovulation. The latter two phases are dependent on stimuli by FSH or LH [60] (Figure 2A).

In humans, and in mice, five stages of follicle development were more specifically described: primordial follicles, primary follicles, secondary follicles (preantral), antral follicles, and preovulatory follicles (Graafian) [117]. Initially, follicular growth is gonadotropin-independent and begins during gestation. These hormones do not directly affect the follicle growth in this phase; however, the granulosa cells (GCs) may be influenced at an earlier stage of preantral follicle growth [61]. The post-natal phases are gonadotrophin-dependent, and the GCs of the large antral to ovulatory follicles are also influenced (Figure 2A). 

During the gonadotrophin-dependent stage of follicular development, FSH and LH signaling pathways play obligatory roles in follicle differentiation, selection, and survival [118]. LH, through the biosynthesis of androgens in theca cells, stimulates the formation of FSHR in GCs and thus potentiate FSH’s effects on secondary and antral follicles. During development of the follicle in the antral follicle phase, a fluid cavity, the antrum, is formed. Antrum fluid is an important source of gonadotropins, steroids, growth factors, and other substances derived from the blood or secretions of the follicular cells (antral folliculogenesis), a process entirely dependent on gonadotropic hormones [119,120] (Figure 2A). 

Antral folliculogenesis occurs in waves of growth and regression; these waves are mediated by levels of FSH to support growth. After the emergence of the dominant follicle, FSH levels decline, the remaining antral follicles regress in a process called atresia, and the dominant follicle will be the only one capable of reaching ovulation with the release of mature oocytes in response to LH pulses [76,121].

During the transition phase of folliculogenesis, the intra-ovarian factors activin and epidermal growth factor (EGF), reported in some species such as pigs [122], cows [123,124], and mice [125], lose importance in follicular growth, and FSH starts acting in the preantral and antral follicle through FSHRs [100,126,127,128,129]. Therefore, when the preantral follicles are formed, the GCs express FSHR and the theca cells express LH receptor (LHR). FSH upregulates cell metabolism and activates the mitogen-activated protein kinase (MAPK) signaling pathways in preantral follicles [130]. In some species such as humans, bovines, sheep, pigs, and rabbits, FSH also regulates preantral follicle development and many paracrine factors from oocytes and granulosa cells [61,127,131,132,133,134].

In addition to the roles of FSH and LH in the follicle, oocyte-secreted growth factors (growth differentiation factor-9 (GDF9) and bone morphogenetic protein 15 (BMP15)) act in follicular maturation, ovulation, fertilization, and luteinization. This interaction between gonadotropin and oocyte paracrine factors is thought to support the physiological mechanism regulating species-specific ovulation rates and fecundity; e.g., in bovines, it is found throughout all follicle development, related to the proliferation and steroidogenesis of granulosa cells [135,136]. In mice, FSH was reported to induce the GCs to express LH receptors and their proliferation in the late stage of follicle formation, and to cause an increase in epidermal growth factor receptor (EGFR) to promote ovulation [137,138]. 

It has been reported in mice that preantral follicles seem capable of development without these hormones, suggesting that gonadotropins are not required for preantral follicular development [139,140]. Recently, it was demonstrated that bovine preantral follicles respond to FSH by upregulating specific cellular functions and pathways [130]. In dogs, it was demonstrated that activin and FSH have a synergistic effect on growth and antral cavitation in both the early and antral stages that is not mediated by changes in FSHR mRNA expression [141].

FSHR is found in some cells in the folliculogenesis process, but the effect of FSH in the initial follicle is unclear [142]. It was shown that mice lacking FSHR are infertile. In humans, single nucleotide polymorphisms (SNPs) in FSHR were reported; however, few of these affect fertility. In women presenting mutations in one of the FSHR genes, follicles grow up to the stage of selectable follicles [143,144]. Interestingly, it was demonstrated that development to the antral phase is not dependent on FSH, as shown in FSH-null mice [145]; preantral follicles are responsive to FSH treatment and, in FSHR and β-FSH gene knockout mice, follicle development occurs until the preantral stage. Studies in mice and rats showed that FSH qualitatively and quantitatively helps with initial follicle development [146], and similar results were found in humans and goats.

Interestingly, some studies in humans and mice reported FSHR expression in oocytes. They discussed, as another role of FSH in ovulation, that it acts in the modulation of meiotic resumption and the completion of oocyte maturation [147,148]. In porcine species, FSHR was observed in oocytes (primary follicles and up to the pre-ovulatory stage) and within the oocyte nests [127].

### 3.2. Males-Influence of FSH in Spermatogenesis

**Figure 3:**
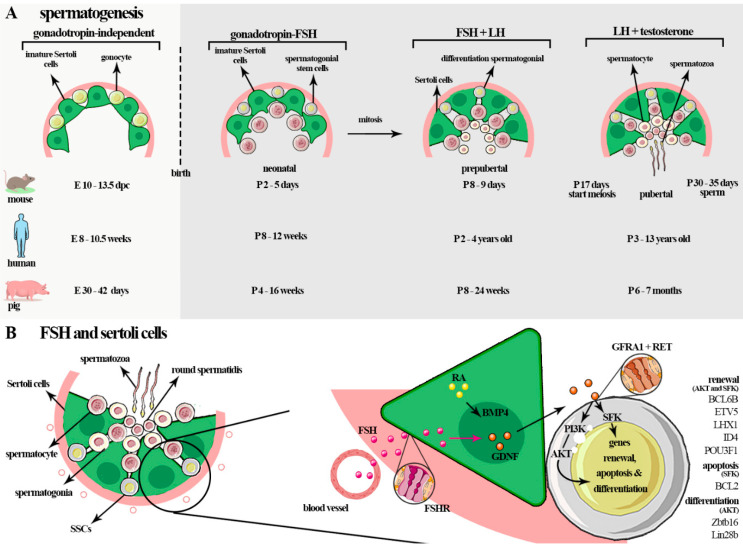
(**A**) In fetal life, PGCs transform into gonocytes that remain centrally placed, surrounded by immature Sertoli cells. In mice, gonocyte development occurs before the formation at E10-13.5 d.p.c, in humans at E 8–10.5 weeks, and in pigs at E30–42 d.p.c (gonadotropin-independent). In the neonatal phase, FSH, through FSHR signaling, regulates the proliferation of the cells and the number of cells that will be had in adult life (~P2–5-day mice (postpartum), ~P8–12 weeks in humans, and ~P4–16 weeks in pigs). During the prepubertal phase, an increase in FSH occurs during the maturation of Sertoli cells and during the completion of the first cycle of sperm (~P8–10 days in mice; ~P2–4 years in humans; ~P8–24 weeks in pigs). In adult life, the spermatogenesis process starts. This is a complex process in which diploid spermatogonia self-renew, proliferate, and differentiate into haploid spermatozoa. The gonadotropins act in the early events of the spermatogenesis, before spermiogenesis, mainly in spermatogonial proliferation and meiosis. These hormones act on all phases of spermatogenesis in some species such as rodents and a specific phase of spermatogenesis in men: the maturation of type A spermatogonia to type B spermatogonia, meiosis, and spermiation. (**B**) In germ cells, FSH mainly influences self-renewal, proliferation, and survival of spermatogonia cells through glial cell-line-derived neurotrophic factor (GDNF) secreted by Sertoli cells. Sertoli cells secrete many factors linked to self-renewal such as GDNF and fibroblast growth factor 2 (FGF2), differentiation and proliferation of spermatogonial stem cells (SSCs), bone morphogenetic protein (BMP4), and activin A, amongst others such as KIT ligand (KL or stem cell factor—SCF), which promotes the KIT tyrosine-kinase receptor expressed by differentiated spermatogonia. GDNF induces SSC self-renew and survival through multiple pathways such as AKT/MEK, AKT, and SFK. The phosphoinositide 3-kinase (PI3K)/AKT pathway influences the self-renewing divisions of SSCs, inhibits apoptosis, and is involved in activating mTORC1 through the SFK signaling pathway. GDNF upregulates the specific SSC genes such as B cell CLL/lymphoma 6, member B (*BCL6B*), Ets variant gene 5 (*ETV5)*, and Lim homeobox protein 1 (*LHX1*). GDNF also acts on the canonical RAS/ERK1/2 pathway, important for the proliferation and self-renewal of these cells by phosphorylation and activation of CREB1, ATF1, CREM, and c-FOS factors.

Spermatogenesis is a sequence of processes during which diploid spermatogonia self-renew, proliferate, and differentiate into haploid spermatozoa [1]. This process occurs gradually with the action of various autocrine, paracrine, and endocrine factors. These factors stimulate multiple events such as the mitotic multiplication and propagation of spermatogonia cells, genetic recombination, and the maturation of spermatozoa [1]. The gonadotropins act on specific phases of spermatogenesis, mainly in maturation (in men during the maturation of type A spermatogonia to type B), meiosis, and spermiation. Notably, the specific effects of FSH or LH on spermatogenesis require more robust characterization [149]. In particular, it is known that FSH acts mainly in Sertoli cells and may support spermatogonia, probably through activating gene transcription related to metabolic homeostasis and cell survival, with the synthesis of retinoic acid, lactate, plasminogen activator type 2, and fatty acid metabolism mitochondrial biogenesis [112,150,151]. Therefore, the entire process depends on hormones, and the gonadotropins FSH and LH play critical roles [105], as shown in Figure 3A.

In humans, FSHβ mutations lead to azoospermia and infertility, although men with mutation in FSHR are phenotypically normal with some subfertility, similar to deficient mice with the same mutation [116]. This difference may be related to genetic and environmental factors. Therefore, few men carrying pathogenic mutations and other possible factors related to the lack or absence of FSHβ and FSHR, which directly affect spermatogenesis, were reported [152]. For this reason, FSH’s action is still not totally understood in human spermatogenesis [1,63]. However, studies in rats reported that FSH is important for the initial spermatogenesis phase, before germ cells start spermiogenesis [153,154].

Studies with animal models suggested that FSH may not be critical to enable fertility; however, it was reported that FSH acts by regulating genes involved in the proliferation, structure, and function of Sertoli cells, being responsible for determining the number of Sertoli cells and their differentiation, possibly by regulating the genes necessary for the metabolism and transport of nutritive and regulatory substances produced by Sertoli cells to germ cells [1,149,155,156]. FSHR signaling in Sertoli cells activates at least five pathways: the cAMP and protein kinase A (PKA) [30], MAP kinase (MAPK), calcium, phosphatidylinositol 3-kinase (PI3K), and phospholipase A2 (PLA2) pathways, which results in the transcription of spermatogenesis target genes [149,157] (Figure 3B).

One of the genes stimulated by FSH is Kruppel-like factor 4 (*KLF4*), important for the differentiation of Sertoli cells during reproductive development, mostly through controlling differentiation and the cell cycle [158]. In addition, FSH, together with testosterone, acts in the Wnt pathway, regulating the inter-Sertoli junction types and the connection between Sertoli cells and germ cells, which allow the cultivation of germ cells [1,113,150,159]. Furthermore, it regulates the expression of genes involved in fatty acid metabolism and mitochondrial biogenesis, which maintains the energy metabolism of seminiferous tubules. Another example of a gene stimulated by FSH is the Aquaporin 8 (*Aqp8*), which is involved in maintaining the water balance of Sertoli cells [160].

Still, regarding the role FSH in male germ cells, it regulates and limits the wave of apoptosis of germ cells during the initial phase of spermatogenesis, which is crucial for maintaining the critical number of cells between some stages of germ cells and Sertoli cells, as the interaction between these two can reduce the efficiency of spermatogenesis [105,161] in addition to increasing spermatogonial differentiation through the glial cell line derived from neurotrophic factor (GDNF) and fibroblast growth factor 2 (FGF2), acquired by Sertoli cells from FSH stimulation. Hence, FSH acts by stimulating spermatogonial proliferation and entry into meiosis [105].

In general, the main effects of FSH were shown to be similar in rodents, primates, and other mammals [149]. However, some differences exist between humans and other primates in terms of sensitivity to FSH by sperm subtypes, and it was observed that type A sperm are more sensitive to gonadotropin suppression in nonhuman primates [110,161]. Additionally, both spermatogonia proliferation and the start of meiosis were shown to be more hormonally sensitive in primates than in rodents, resulting in a greater need for FSH in primates to ensure fertility [160]. Rodents can complete spermatogenesis without FSH stimulation, but its deficiency significantly decreases sperm quantity. Xenotransplantation of canine spermatogonial stem cells (SSCs) into mice testicular tissues was found to promote spermatogenesis in infertile mice when regulated by FSH [162].

## 4. Influence of FSH on Germ Cells

This section discusses the role of FSH in GCs and Sertoli cells and, indirectly, in germ cells in mammalian males. For more than 10 years, the specific action of gonadotropins in these cells has been discussed, mainly focusing on which pathways and genes are activated during folliculogenesis and spermatogenesis; however, few in vivo studies have addressed the role of FSH in germ cells as they constitute the population of undifferentiated spermatogonia.

### 4.1. Females: Granulosa Cells

In mammalian females, FSH binds to specific transmembrane receptors (FSHRs) located on the membrane of the granulosa cells (GCs) of developing follicles, and, consequently, affects fertility [44]. The granulosa cells arise during the primordial follicle phase arise as flat cells, through the epithelial–mesenchymal transition, surrounding the oocyte. GCs are the only somatic cells that closely interact with the oocyte from the moment of follicular formation until ovulation [163]; therefore, they are the first cell type in the ovary that provides an adequate environment with the physical and chemical conditions for oocyte development and maturation [164].

FSH acts in folliculogenesis, including in the induction of GCs proliferation [165,166] and stimulation of GCs estrogen (i.e., estrone and estradiol) production, via aromatase (CYP19A1) conversion of the androgens produced by theca cells (i.e., dehydroepiandrosterone (DHEA), androstenediol, androstenedione, and testosterone) from cholesterol in response to LH [163,167]. Physiological responses to FSH during this process occur by activating multiple signaling pathways that regulate the expression of specific target genes [168,169,170]. In pigs, it was shown that FSH regulates several genes in vitro when GCs were analyzed [171]. In pre-antral granulosa cells, the activation of protein kinase A (PKA), which is a major regulator of transcription factors, showed dependency on FSH to drive the differentiation of granulosa cells (Figure 2B) (reviewed in [168]).

Recently, a study in mice showed that FSH can be involved in ovarian cell development through the regulation of *OCT4* expression in granulosa cells at the preantral to early antral transition stage of follicles via the glycogen synthase kinase (GSK)-3β/β-catenin pathway, and this response was found to be mediated by the phosphoinositide 3-kinases/serine/threonine kinase (PI3K/Akt) signaling pathway [172]. In many species, GCs from preantral (at the secondary follicle stage) and antral follicles at different stages of follicular growth were shown to express FSHR [126,127,128].

The interaction of FSH–FSHR promotes the differentiation of GCs and the maturation of follicles [173]. Specifically, FSH connects to the FSHR on the surface of GCs, activates adenylyl cyclase (AC), and stimulates the proliferation of GCs by activating the cyclic adenosine monophosphate/protein kinase A (cAMP/PKA), mitogen-activated protein kinase/extracellular signal-regulated kinase (MAPK/ERK), and PI3K/Akt pathways. In particular, cAMP/PKA promotes the phosphorylation of the cyclic AMP response element binding (CREB) protein and other proteins that promote increased expression of genes that encode growth factors and proteins involved in steroid hormone production and cellular growth. This gonadotropin, via PI3K/Akt pathway, can also impede the apoptosis of GCs [30,174,175]. However, the mechanism through which FSH stimulates the differentiation of the cells is still unclear (Figure 2B).

The granulosa cells (GCs) from primary and preantral follicles secrete anti-Müllerian hormone (AMH), which acts in folliculogenesis mainly by decreasing the sensitivity of preantral follicles to FSH and, consequently, decreases the aromatase expression promoting dominant follicle selection. AMH also inhibits the primordial follicular growth of the ovarian reserve, thus negatively regulating the follicle growth [142]. The evidence of the AMH effect was confirmed by administration of FSH in pre-pubertal AMH (−/−) knockout mice, increasing the follicle growth rate [176]; similar results were reported in humans when AMH was added to an in vitro culture of GCs [177]. AMH was shown to be present at high levels in puberty once this period presented high rates of oocyte maturation and at low levels in menopause [178]. Other pathways have been reported to control the regulation of follicular and oocyte development, e.g., the BMP-SMAD1/5/8 pathway is essential in follicular activation and development, GCs proliferation, atresia, and luteinization, and the Notch pathway is activated by gonadotrophins, which is important in oocyte development [179,180,181].

As reviewed [118], the GCs of early antral follicles are stimulated by FSH, which induces LH receptor formation on GCs from the pre-ovulatory follicle. Then, the maturing follicle reduces its dependency on FSH by acquiring LH receptors and LH responsiveness. Without the stimulation of FSH, the follicle does not develop and atresia occurs [126]. In women and cows, differently from other species such as mice, only a single follicle is selected to ovulate every cycle; and this selected (dominant) follicle phase surges after the FSH peak. The dominant follicles (DF) are at the more advanced stage of maturation, they are growing, and more FSH-sensitive. The transition from FSH- to LH-dependence is considered necessary for their continued development and further ovulation after LH peak [117,182,183].

Recently, researchers have discussed that FSH action on GCs can be influenced by androgens, such as testosterone or dihydrotestosterone. In human and non-human primates, these androgens positively impact granulosa cell stimulation by FSH. Furthermore, androgens were shown to significantly increase the amounts of FSHR mRNA in the preovulatory follicles of post-pubertal pigs. In addition, FSHR may modulate and impact cell death signals in ovarian cells [30,184,185].

Other cells are indirectly stimulated by FSH; for example, cumulus cells (COCs) and very small embryonic-like stem cells (VSELs). During the transition phase from the preantral to the antral follicle, the differentiation of granulosa cells into cumulus cells depends on oocyte signals and the indirect action of FSH. An in vivo study reported the importance of FSH endocrine in the cumulus expansion and cumulus cell signaling network through epidermal growth factor (EGF) and the FSH-CAMP-PKA pathways. One of these studies was performed with FSHβ −/− mice and showed that these animals were unable to form large antral follicles or express epidermal growth factor receptor (EGFR) in the disturbed follicle, promote meiotic oocyte progression, or differentiate cumulus cells. However, all of these functions were recovered when the follicles were exposed to exogenous FSH [138]. Furthermore, exposure of porcine COCs from antral follicles to exogenous FSH promoted EGF induction via EGFR [186], a fundamental factor in oocyte maturation, cumulus expansion, and ovulation. However, Richani and Gilhris [187] stated that this FSH interaction alone is insufficient, and other interactions between hormone signaling and paracrine oocyte signals are necessary to influence EGF functionality in COCs.

Bhartiya and Singh [188] discussed the regulation of FSH–FSHR on ovarian stem cells (OSCs) localized on the surface of ovarian epithelial cells (OSE). OSEs were reported to comprise two distinct cell populations of stem cells: very small embryonic-like stem cells (VSELs) and ovarian stem cells (OSCs). They are responsible for the neogenesis and assembly of the primordial follicle in adulthood [189,190,191]. VSELs, together with OSCs/ovarian germline stem cells (OGSCs), have been found in mouse, rabbit, sheep, monkey, and human ovaries [188,190,192,193]. VSELs and OSCs are understudied cells that have generated considerable discussion; however, cells are capable of self-renewal and giving rise to ovarian stem cells (OSCs), which rapidly divide, form germ cell nests, and differentiate into oocytes [188,194].

FSH modulates the VSEL and OSC populations. According to Patel et al. [195], FSH exerts direct action through the expression of FSHR3 in these cells via the MAPK/ERK pathway (suggesting the involvement of FSHR3). This FSHR3-mediated action of FSH on OSE was implicated in ovarian biology as well as pathology [195]. FSHR3 (specifically in exon 11) is one of the four known alternative splicing isoforms of FSHR (R1, R2, R3, and R4) [196]. Specifically, FSHR3 is a fundamental and abundantly expressed transcript related to follicular processes and ovarian cancer cells. Studies showed that high levels of FSH gonadotropin or mutation in FSH-FSHR3 in OSE may result in menopause, and are most probably responsible for various pathologies such as premature ovarian failure (POF) and cancer [188]. Therefore, with the elevated FSH level associated with advancing age, the somatic microenvironment is compromised. Thus, the oocytes do not differentiate, and senescence occurs (menopause in women). In this situation, OSE can undergo uncontrolled proliferation, resulting in ovarian cancer [197]. More detailed studies are still needed to better understand ovarian biology, the influence of FSH in the niche of cells that results in menopause, and pathologies with cancer and premature ovarian failure.

### 4.2. Males: Sertoli Cells and Spermatogonial Stem Cells (SSCs)

Sertoli cells are the only cell in the seminiferous tubules that present FSH receptors (FSHRs); through these, FSH controls the function of these cells, thereby exerting indirect action in spermatogenesis modulation, germ cell survival, and male fertility. The Sertoli cells are essential for the spermatogenesis process, providing nutritional and structural support for functional germ cells. They are fundamental to protecting germ cells through the blood–testis barrier (BTB) and through the production of immunomodulatory factors [1]. In addition, Sertoli cells control the germinal stem cells niche and create an adequate microenvironment for germ cell development [112] (Figure 3B). 

During the prepubertal phase in rodents, Sertoli cells mature and the first cycle of spermatogenesis is completed; these events are associated with an increase in FSH secretion [113]. To confirm the influence of FSH on germ cells, a study in mice showed that the lack of FSH or FSHR does not lead to azoospermia or sterility; however, it decreases testis size and the number of germ cells, mainly spermatocyte and spermatogonia cells, to <50% [198,199]. This occurs because the lack of FSH reduces the Sertoli cell number and the capacity to support and nurture germ cells [1]. In primates, for example, humans, the abolition of FSH secretion or action will not prevent either the initiation or the maintenance (qualitative) of spermatogenesis and, therefore, does not lead to azoospermia. However, the quality of the sperm produced in the absence of FSH remains to be determined [110].

Ruwanpura et al. [149] reviewed the influence of FSH on spermatogonial and other types of germ cells (spermatocytes and spermatids) in rodents and humans. The authors stated that FSH has little effect on the proliferation of germ cells in vivo compared to in vitro, which may be related to a low concentration of FSH. However, studies with transgenic rats and mice showed that the FSH level regulates the survival of the spermatogonial population and spermiation process [200]. In rats, changes in FSH level may result in a time-dependent change in the differentiation of the number of type A/intermediate spermatogonia [200], mainly with the A3–A4 spermatogonia in stages XIV–I [201] and specific stages of the seminiferous tubules (XIV–III and VI–VIII) [202]. 

In primates, two morphologically distinct types of undifferentiated spermatogonia were described in the testes of Rhesus macaques, and these cells were designated A_1_ and A_2_, later renamed A_dark_ and A_pale_, respectively, representing the reserve and renewing stem cells, respectively [203,204,205,206,207]. The shift in the number of spermatogonial type A to type B spermatogonia and alteration in the proliferation of spermatogonial type A_pale_ were observed when gonadotrophin was suppressed [208,209]. Studies in humans showed that FSH suppression for 12 weeks might promote the decrease in spermatogonia type B (10–20% of control) and type A_pale_ (40%) numbers [210].

In other germ cells, such as spermatocytes, the role of FSH is still not entirely clear [149], and studies suggest that FSH may have a role in meiosis and spermiogenesis, affecting sperm population [149,159]. According to Eto et al. [211,212], in murine testes, the possible actions of FSH in the progress of meiosis occur via nociceptin/OPRL1 (Opioid Related Nociceptin Receptor 1), which is upregulated via cAMP/PKA/CREB. They suggested that the high FSH level stimulated Sertoli cells to produce nociceptin (neuropeptide), which binds to nociceptin receptor (OPRL1), localized in the plasma membrane of spermatocytes, to promote REC8 phosphorylation, which is responsible for meiotic chromosomes during meiosis. In addition, FSH, together with retinoic acid (RA), promotes meiosis through the induction of Sertoli cells expressing the neuregulin 1 (NRG1) and NRG3 with their receptor protein-tyrosine kinase ErbB-4 (ERBB4) in pre-spermatocytes, indicating the possible action of FSH in the progression of meiosis via nociceptin/OPRL1 [213].

FSH might support meiosis in mice by maintaining preleptotene spermatocytes and partially maintaining pachytene spermatocytes; the absence of FSH may reduce stage VII pachytene spermatocyte numbers when the androgenic level is normal [201]. In humans, however, FSH alone appears not to play an essential role in the processes of meiosis and spermiogenesis; it must act alongside LH in the maintenance of spermatocytes. Similar to that described in rodents, FSH and testosterone act as survival factors for spermatocytes and spermatids [214]. In conclusion, the studies showed that FSH is essential for first-wave spermatogenesis (it is able to support mitosis and meiosis in this phase), survival, and self-renewal of the spermatogonial population. However, FSH together with other factors, such as androgen, LH, or testosterone, is necessary to complete meiosis and spermiogenesis.

Currently, it is accepted that the biological effect of FSH may occur through the FSHR found in Sertoli cells, which stimulates these cells to secrete factors that regulate undifferentiated spermatogonia, promoting the self-renewal and maturation of these cells. Specifically, Sertoli cells secrete many factors linked to self-renewal such as glial cell-line-derived neurotrophic factor (GDNF) and fibroblast growth factor 2 (FGF2); and differentiation and proliferation of spermatogonia stem cells (SSCs) such as bone morphogenetic protein (BMP4), activin A, and others, such as the tyrosine-kinase receptor c-kit ligand, (stem cell factor—SCF or KIT ligand—KL), in response to FSH stimulus. Also, germ cells may generate signals that control, locally, the balance of GDNF vs. BMP4 and KL [7] (Figure 3B).

GDNF is the paracrine factor responsible for the maintenance and self-renewal of SSCs, and the differentiation of spermatogonia is inhibited by activating the zinc finger BTB domain containing 16 (*ZBTB16*) and Lin-28 homolog B (*LIN28B*) [215,216,217]. In particular, FSH connects with the FSHR in the membrane of Sertoli cells that secrete GDNF, which, in turn, acts via RET tyrosine kinase, which requires a ligand-specific co-receptor GDNF family receptor alpha 1 (GFRα1), localized in the membrane of undifferentiated spermatogonia to promote self-renewal [216,217,218,219,220]. This GDNF–RET connection activates the tyrosine 1062 pathways, which are fundamental to the self-renewal process [221]. GDNF-induced SSC self-renewal and survival occurs through multiple pathways such as AKT/MEK, AKT, and Src family kinase (SFK) [222]. The MEK signaling pathways in SSCs promote the increased generation of reactive oxygen species (ROS) generated by NADPH oxidase 1 and stimulate SSC proliferation and self-renewal through the activation of p38 and JNK MAPKs [223]. This was shown in mice; GDNF signaling is fundamental to maintain NANOS2 expression in SSCs. NANOS2 is important for inhibiting meiosis in fetal gonocytes, and preventing spermatogonial differentiation in the post-natal testes [224,225] (Figure 3B).

Recently, it was found that the effect of GDNF on SSCs is related to the activation of AKT and the Src family kinase (SFK) signaling pathway [226]. The phosphoinositide 3-kinase (PI3K)/AKT pathway influences the self-renewing divisions of SSCs and inhibits apoptosis; it is also involved in the activation of mTORC1 [227,228] through the SFK signaling pathway. GDNF upregulates specific SSCs genes such as B cell CLL/lymphoma 6, member B (*BCL6B*), ETS variant gene 5 (*ETV5*), Lim homeobox protein 1 (*LHX1*), DNA-binding protein 4 (*ID4*), Brachyury (*T*), and POU class 3 homeobox 1 (*POU3F1*) [226,229]. Some of these transcription factors, such as *BCL6*, *EVT5*, *ID4*, *FGFR1*, and *RET*, were found to be highly expressed in gonocytes and SSCs from mice and SSCs from prepubertal human testes [230]. GDNF also acts in the canonical RAS/ERK1/2 pathway, which is important for the proliferation and self-renewal of these cells by phosphorylation and activation of CREB1, ATF1, CREM, and c-FOS factors [226,231,232,233]. Overall, it is clear that active signaling of GDNF in vivo mainly acts in the maintenance of the undifferentiated state of SSCs and, in vitro, stimulates the proliferation of immature Sertoli cells [234] (Figure 3B).

In mouse testes, the highest number of undifferentiated spermatogonia was observed after the overexpression of GDNF [235], which promotes self-renewal of SSCs, whereas a lower level stimulates the differentiation of these cells [216,236]. Furthermore, heterozygotic-GDNF-deficient mice are fertile. In addition, it was found that spermatogenesis deteriorates with age as germ cells deplete, similar to what is observed in humans. This suggests that GDNF can also influence the differentiation of SSCs [216], suggesting that GDNF also influences the differentiation of SSCs. 

In other species, such as canines, FSH might affect these cells in vitro and in vivo. In one study, the authors found that canine SSC (cSSCs) numbers increased in vitro in the presence of FSH. When cSSCs were transplanted in chemoablated mouse testes, cSSCs were found in seminiferous tubules. We discussed this paracrine effect of FSH as being possible via Sertoli cells, which express FSHR and secrete more GDNF in the presence of FSH, resulting in increased numbers of SSCs [162]. Other in vitro studies with bovine and mouse SSCs demonstrated the influence of FSH in the self-renewal and proliferation processes mediated by GDNF and FGF2 [237], confirmed by the expression of GFRα1 [238,239].

## 5. Influence of FSH in Germ Cells In Vitro

Studies in animals and humans showed that FSH may positively affect the germ cells of males (Sertoli and spermatogonial stem cells) and females (oocyte and follicle) in vitro. In this environment, FSH may facilitate the development of the antral follicle, and increase oocyte quality and SSCs proliferation. In addition, this hormone supports the differentiation of primordial germ-cells-like cells (PGCLCs) into mature germ cells. Hence, we herein describe some studies that used FSH to create an in vitro environment similar to the in vivo environment.

FSH acts on follicular development and growth in females; a role is also played in in vitro models during the early stages of folliculogenesis [2,240,241]. However, the effects of FSH in culture may vary according to several factors, such as FSH source and concentration, species, and even culture system (reviewed by [213]). In humans, low doses of FSH associated with activin were shown to increase oocyte quality [242], whereas, in other non-human primates (Rhesus monkeys), the absence of FSH in in vitro culture resulted in non-surviving secondary follicles [243], unless the medium was supplemented with ovarian steroid hormones [244]. Amongst domestic species, FSH has presented diverse effects during in vitro culture. In bovines and caprines, media containing FSH supplementation combined with other factors increased oocyte maturation rates [241,245,246,247].

During in vitro culture and maturation of canine oocytes, however, Lee et al. found that FSH increases cumulus cells expansion and affects nuclear maturation rates, but not with the same intensity as other domestic species [248]. Hu et al. reported increased numbers of metaphase I and metaphase II oocytes when in vitro maturation media were supplemented with FSH, estradiol, and progesterone; however, differences among groups were not significant [249]. Furthermore, in domestic cats, higher oocyte maturation rates were detected in those cultured with an association of FSH, LH, and estradiol [250].

In males, some in vitro models were used to determine the influence of FSH in the spermatogenesis [26], survival, proliferation, and self-renewal of spermatogonial stem cells (SSCs) [6]. The induction and maintenance of spermatogenesis are multihormonal-pathways-dependent; in addition, FSH, testosterone, and LH play essential roles in this process [6,251].

In vitro spermatogenesis showed dependence on FSH and used a co-culture of Sertoli cells and SSCs [6]. In association with testosterone, FSH prevents human germ cell apoptosis [252]; the Sertoli cells become able to bind round spermatids by FSH. Tesarik and collaborators [253,254] showed that a high concentration of FSH in an in vitro culture is associated with the morphological changes presented in round spermatids in humans. Furthermore, FSH stimulates meiosis II and late spermatid differentiation, and, in those processes, testosterone can potentialize FSH’s action [255].

In mice, the proliferation of Sertoli cells in vitro and the mitosis and meiosis events in germ cells development were promoted with FSH stimulation in the absence of LH [256]. Likewise, in humans, the authors [257] demonstrated that FSH and testosterone positively affect meiotic division and the reduction in germ cell apoptosis. FSH supplementation in an in vitro culture of cSSCs promoted an increased rate of proliferation and self-renewal, confirmed by the increased numbers of GFRα1-positive cells (receptor of GDNF in SSCs) and the formation of germ cell clumps in vitro [162]. In vitro studies in animal models on the effect of FSH in male species other than dog [162] or mouse/rat [1,159,258] are still needed.

Primordial germ-cells-like cells (PGCLCs) were generated in vitro from both iPSCs and ESCs, even generating viable offspring in mice [259]. This methodology is a significant advancement in the field of assisted reproduction and animal preservation [162], and has been applied to different species, such as mice [259,260,261], humans [262,263], nonhuman primates [264], pigs [265], and goats [266], although more robust results were achieved in mice. Supplementation with FSH, bovine pituitary extract, and testosterone resulted in an increased percentage of mouse ES-derived PGCLCS completing meiosis in vitro, resulting in spermatid-like cells (SLCs) [267]. In humans, supplementation with FSH associated with growth factors and other hormones resulted in ESCs/iPSCs-derived SSCLCs capable of in vitro propagation and differentiation into spermatocytes and haploid cells [262]. Furthermore, FSH supplementation during in vitro growth and maturation was used for the generation of mouse iPSC- and ESC-derived oocytes in vitro [268] (Table 1).

## 6. Conclusions

For decades, the role of gonadotropins during reproductive life has been discussed, mainly concerning the signaling pathways and genes that are regulated by these hormones. Technological advances have allowed us to further understand the role of hormones in germ cells, although many studies have been conducted in rodents. As discussed in this review, gonadotropins (LH and FSH) play a key role in fertility. In both sexes, FSH exerts its action through receptors (FSHR) located on target cells in the testes and ovaries (Sertoli and granulosa cells). Some studies even found that these receptors can also be detected in other cells of extra-gonadal tissues such as VSELs. 

FSH begins its activity after birth in females, although FSHR has been detected in primary follicular cells. This role is intensified in adult life, when this hormone, through FHSR located in GCs, promotes follicular growth and maturation, acting on pre-antral and antral follicles. Therefore, women and rodents deficient in FSHRs present a blockage in the production of follicles, becoming infertile. In mammalian males, FSH directly influences Sertoli cell proliferation during fetal and neonatal life and, consequently, spermatogenesis and sperm production in adulthood. These cells are the main target of FSH in the seminiferous tubules. Thus, through FSHR located in the membrane of Sertoli cells, FSH binds to these cells and stimulates the release of factors that help in self-renewal, such as GDNF and FGF2, and the differentiation (BMP4, activin A, and KL) of SSCs. Therefore, FSH deficiency in mice and humans can reduce spermatogenesis and azoospermia in some cases. In vitro, some studies with SSCs proved the influence of FSH in these processes in the germline. Recently, it was shown that the addition of FSH with other factors (such as testosterone) could promote the differentiation of PGCLCs into mature gametes in vitro. 

## Figures and Tables

**Table 1:** The use of FSH in vitro to SSCLCs generation and oocyte maturation from ESCs or iPSCs cells.

Species	Cell Type	FSH Supplementation	Outcome	Author
Mouse	ESCs and iPSCs	FSH on in vitro growth media and in vitro maturation media	Mature oocytes/viable offspring	[259]
Human	ESCs and iPSCs	Recombinant human FSH in SSCLCs generation media	Human spermatogonial stem cells, further differentiation into spermatocytes and haploid cells	[262]
Mouse	ESCs	Spermatogenesis induction media contains FSH, testosterone, and bovine pituitary extract	Haploid spermatid-like cells/viable offspring	[267]

## Data Availability

Not applicable.
